# Differential Effects of Roads and Traffic on Space Use and Movements of Native Forest-Dependent and Introduced Edge-Tolerant Species

**DOI:** 10.1371/journal.pone.0148121

**Published:** 2016-01-28

**Authors:** Hsiang Ling Chen, John L. Koprowski

**Affiliations:** School of Natural Resources and the Environment, The University of Arizona, Tucson, Arizona, United States of America; Institute of Agronomy, University of Lisbon, PORTUGAL

## Abstract

Anthropogenic infrastructure such as roads and non-native species are major causes of species endangerment. Understanding animal behavioral responses to roads and traffic provides insight into causes and mechanisms of effects of linear development on wildlife and aids effective mitigation and conservation. We investigated effects of roads and traffic on space use and movements of two forest-dwelling species: endemic, forest-dependent Mount Graham red squirrels (*Tamiasciurus hudsonicus grahamensis*) and introduced, edge-tolerant Abert’s squirrels (*Sciurus aberti*). To assess the effects of roads on space use and movement patterns, we compared the probability that a squirrel home range included roads and random lines in forests, and assessed effects of traffic intensity on rate of road crossing and movement patterns. Red squirrels avoided areas adjacent to roads and rarely crossed roads. In contrast, Abert’s squirrels were more likely to include roads in their home ranges compared to random lines in forests. Both red squirrels and Abert’s squirrels increased speed when crossing roads, compared to before and after road crossings. Increased hourly traffic volume reduced the rate of road crossings by both species. Behavioral responses of red squirrels to roads and traffic resemble responses to elevated predation risk, including reduced speed near roads and increased tortuosity of movement paths with increased traffic volume. In contrast, Abert’s squirrels appeared little affected by roads and traffic with tortuosity of movement paths reduced as distance to roads decreased. We found that species with similar body size category (<1 kg) but different habitat preference and foraging strategy responded to roads differently and demonstrated that behavior and ecology are important when considering effects of roads on wildlife. Our results indicate that roads restricted movements and space use of a native forest-dependent species while creating habitat preferred by an introduced, edge-tolerant species.

## Introduction

Global biodiversity is threatened by human-induced changes in the environment [1,2]. Anthropogenic development and non-native species are major causes of species endangerment [3,4]. Linear infrastructure, including roads, railways, and power line corridors are undoubtedly widespread and significant artificial features on the planet [5,6]. The ecological effects of roads are diverse and substantial. Road construction not only causes destruction and loss of habitat but also facilitates deforestation and landscape fragmentation [7]. Roads and traffic influence wildlife populations directly through mortality due to wildlife-vehicle collisions, and indirectly by changing animal behavior via visual and auditory disturbances [8,9]. Furthermore, roads facilitate human access, hunting and poaching as well as introduction and establishment of exotic species [5,7,10]. Introduced species rank as the second most prevalent cause of extinction and endangerment of species, and negatively impact native species through predation, resource competition, hybridization and disease transmission [3,11–13]. Roads enhance invasion and spread of exotic species (e.g. *Lythrum salicaria* [14]; *Bufo marinus* [15]) by altering roadside environment and facilitating movements [7,16]. Impacts of roads on population density and community structure due to changes in vegetation structure and microclimate can extend several kilometers from the road [17–21].

Understanding behavioral responses of animals to roads and traffic provides insight into causes and mechanisms of effects of linear development on wildlife and aids effective mitigation and conservation [22,23]. Responses to roads and traffic likely vary considerably across species [20,24]. For some taxa, roads and traffic serve as barriers that impede movements and decrease accessibility of resources, but facilitate movements and increase foraging habitat of others [7,25–27]. Such differences in behavioral response to roads may further affect interspecies relationship such as competition between native and introduced species. Tree squirrels (*Sciurus* and *Tamiasciurus*) are an ideal group for investigating the effects of roads. Arboreal squirrels are widespread, common, and are readily sampled and tracked by radio telemetry because of moderate home range size [28,29]. Moreover, tree squirrels show a spectrum of sensitivity to habitat fragmentation [30]. Yet, the specific impacts of roads on tree squirrels, and how these impacts may differentially affect sympatric species are mostly unknown.

One fundamental question in road ecology is how animals respond to vehicles and traffic disturbance. However, few studies have examined the effects of roads and traffic on animal movements beyond road crossings. Traffic volume is suggested to have little effect on the rate of road crossing by small mammals, and increasing traffic volume did not decrease the success of return by small rodents after translocation [31,32]. Nevertheless, animals may cross high traffic roads during low traffic periods, with the result in animal space use that is similar between high and low traffic roads [32]. Besides influencing the rate of road crossing, traffic may affect animal movement patterns near roads, including animal distance from roads, speed, and path tortuosity. Fine scale records of traffic and animal movements are required to further understand effects of traffic intensity [33]. Herein, we investigated and compared effects of roads and traffic on space use and movements of two forest-dwelling species: endemic, forest-dependent Mount Graham red squirrels (*Tamiasciurus hudsonicus grahamensis* [34]) and introduced, relatively edge-tolerant Abert’s squirrels (*Sciurus aberti* [35]). We examined the spatial relationship between squirrel home ranges and roads, and assess whether roads represent barriers to animal space use. We investigated effects of daily and hourly traffic volume on rate of road crossing and fine-scale movement patterns, and compared observed results with simulated correlated random walks. Because roads may serve as barriers for native forest-dependent species, and inhibit their access to food or other resources critical for survival, while simultaneously providing habitat for exotic edge-tolerant species [10,15,36], a better understanding of species-specific responses to roads and traffic will permit evaluation of effects of human linear infrastructure on the relationship between native and exotic species to assist management.

## Methods and Materials

### Study area and study species

**Fig 1:**
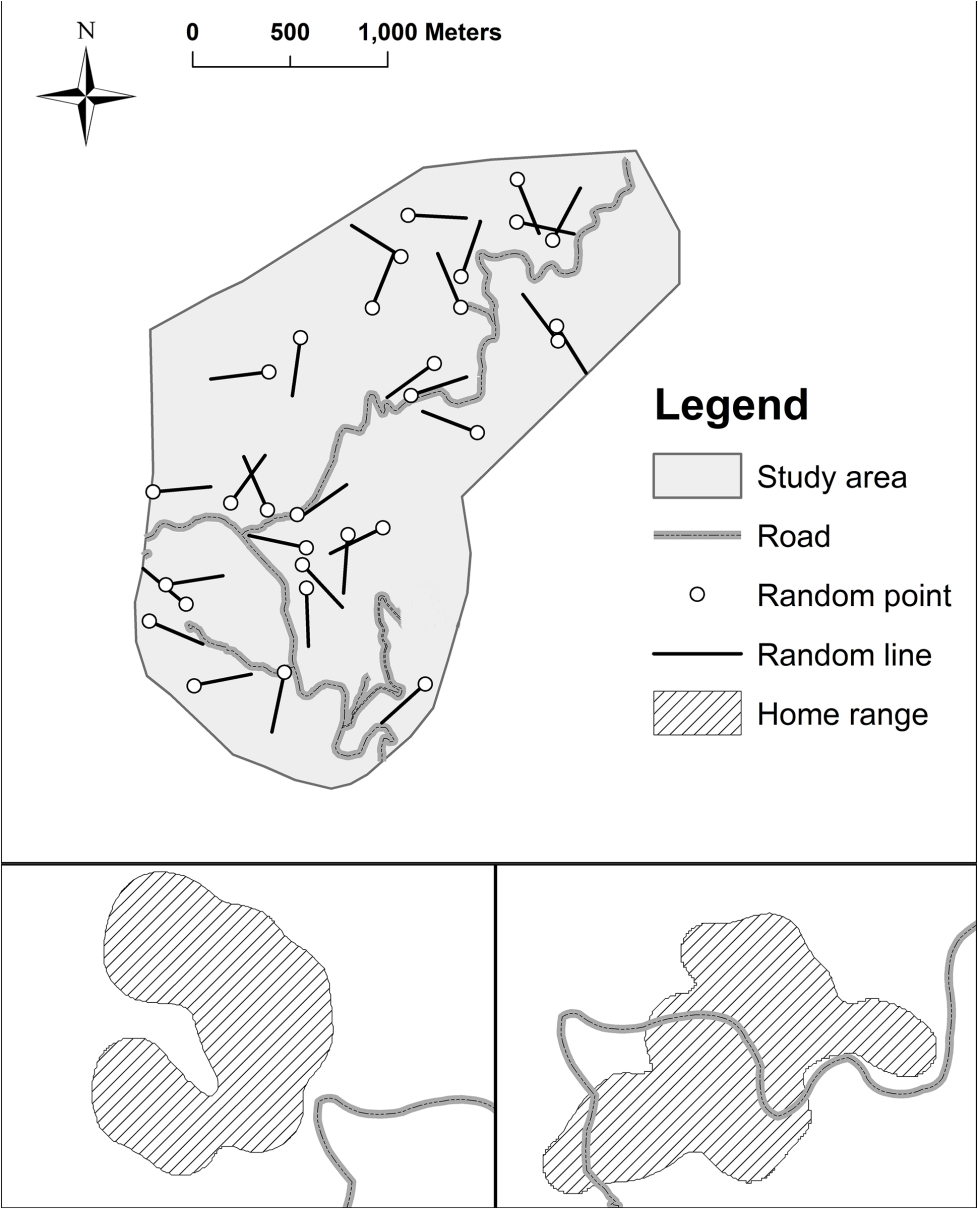
Maps of study area, roads, and random lines (Fig a) and examples of a home range of squirrels that included roads (Fig b) and did not include roads (Fig c), Mt. Graham, Arizona, USA.

Our study was conducted in 546 ha of mixed-conifer forest >3,000 m elevation in the Pinaleño Mountains (Graham Mountains), Graham County, Arizona, USA (32° 42′ 06″ N, 109° 52′ 17″ W). We focused on 10 km along four graded dirt roads, which were the only roads in our study area (Fig 1a): Arizona State Highway 366 also known as Swift Trail (6 to13-m wide, annual average daily traffic [AADT]: 50 vehicles, hereafter, high traffic), the access road to the Mount Graham International Observatory (4 to10-m wide, AADT: 23 vehicles, hereafter, medium traffic), the Bible Camp Road (4 to 9-m wide, AADT: 25 vehicles, hereafter, medium traffic), and Soldier Trail (3 to 24-m wide, AADT: 7 vehicles, hereafter, low traffic). Speed limit on all roads was 40 km/h. Roads were closed to the public from 15 November to 15 April annually. No wildlife road crossing structures were installed in the study area. The forest was dominated by Douglas-fir (*Pseudotsuga menziesii*), southwestern white pine (*Pinus strobiformis*), and corkbark fir (*Abies lasiocarpa* var. *arizonica*) interspersed with Engelmann spruce (*Picea engelmanii*), aspen (*Populus tremuloides*) and ponderosa pine (*Pinus ponderosa*) [37].

The North American red squirrel is a small (<300 g), diurnal tree squirrel with a wide-ranging distribution in Canada and the United States [38]. Red squirrels are larderhoarders and rely on cone-scale piles known as middens to store food for survival, especially in winter [38,39]. Red squirrels are territorial and center their territories on middens [38,39]. Middens are typically located in forests with dense canopy (~90%) and understory cover and provide a cool and moist microclimate that prevents cones from opening and releasing seeds [34,40,41]. The Mt. Graham red squirrel is a subspecies that is isolated and endemic to high elevation forests (>2,000 m) of the Pinaleño Mountains, which are surrounded by desert and grassland, and harbors the southernmost population of red squirrels [38,42]. Because of geographic isolation, low population numbers (~ 300 individuals [37]), and habitat destruction, the Mt. Graham red squirrels were listed as federally endangered in 1987 [43]. Abert’s squirrels are large (600–900 g), diurnal, non-territorial tree squirrels [44]. In the United States, Abert’s squirrels are native to mountains in the southwestern U.S., but were not present on Mt. Graham before introduction in 1941–43 [42,45,46]. Abert’s squirrels were thought to be a ponderosa pine obligate [47]; however, red squirrels and Abert’s squirrels are present in mixed-conifer and spruce-fir forests on Mt. Graham [48] and have similar diets focused on conifer seeds and fungi [49]. Unlike red squirrels, Abert’s squirrels are scatter holders and known to remove cones from red squirrel caches at middens [50–52]. Because of considerable dietary overlap between red squirrels and Abert’s squirrels [38,49], introduced Abert’s squirrels can decrease long-term viability of endangered native red squirrels through food competition [53,54]. An additional potential threat to Mt. Graham red squirrels is human disturbance from recreation, road traffic, and habitat modification associated with road improvement [41,55,56]. Because Abert’s squirrels are more terrestrial, and select more open nest sites (~80% canopy cover) than those of red squirrels (~86% canopy cover [35,52,54]), Abert’s squirrels may respond to roads differently from red squirrels.

### Effects of roads on animal space use

#### Radio telemetry

To track red squirrels and Abert’s squirrels, we used standard methods [29,57] to trap, affix unique ear tags and fit radio collars on squirrels. We placed traps at active middens of red squirrels and where feeding signs of Abert’s squirrels present in entire study area to maintain a population of marked animals. From 2011 to 2013, we captured and collared 100 red squirrels (57 adults and sub adults, 43 juveniles) and 33 adult Abert’s squirrels, and these correspond to all known individuals. From June to November in 2011, 2012 and 2013, we located squirrels during daylight hours via simultaneous biangulation and homing [29]. Locations were taken >60 min apart to ensure independence. The proportion of homing locations was less than 5% for red squirrels and was 63% for Abert’s squirrels. We recorded behavior when squirrels were visible. We used data collected by radio telemetry to estimate seasonal 95% (total) and 50% (core) fixed kernel home ranges for individual red squirrels and Abert’s squirrels each summer (June to August) and fall (September to November [29]) annually. We used least-squares cross-validation (LSCV) to select bandwidths [29]. Home ranges estimated with <15 fixes were excluded. Mean number of locations was 35 fixes (SE 0.9, *n* = 119). Mean total (95%) home range size was 3.6 ha (SE 0.6) for red squirrels and was 11.9 ha (SE 1.0) for Abert’s squirrels. We estimated the center of total and core home ranges with the Geospatial Modelling Environment (GME [58]). Field efforts were conducted under permits from the United States Department of Agriculture Forest Service, Arizona Game and Fish Department, United States Fish and Wildlife Service, and the University of Arizona’s Institutional Animal Care and Use Committee (Protocol #11–248).

#### Data analysis

To assess effects of roads on space use by squirrels that were resident nearby, we used high-resolution aerial imagery obtained from the National Agriculture Imagery Program (NAIP) in 2007 to digitize roads, and used an approach on the basis of a previous study [59] to create random lines in forests to serve as a control linear category. To create random lines in the forests with similar density of roads (1.73 km/km^2^), we first used ArcGIS Desktop 9.3 (Environmental Systems Research Institute) to produce 30 random points and then generated 300-m straight lines from each point in a randomly selected direction (Fig 1a). To investigate if red squirrels and Abert’s squirrels select or avoid areas adjacent to roads, we recorded two types of responses: (1) distance from the center of total (95%) and core (50%) home ranges to roads and random lines, and (2) whether total and core home ranges of squirrels include any roads and random lines (Fig 1b and 1c). To avoid the situation that roads or random lines are too far to be included in home ranges of squirrels, we only used data for red squirrels that occupied middens <100 m from any random lines and roads and data for Abert’s squirrels that were captured <200 m from random lines and roads. We based 100 m and 200 m on size of home range and mobility of red squirrels [29] and Abert’s squirrels [57] respectively. During natal dispersal, movement patterns of juvenile red squirrels differ from adults [60]. Therefore, we only included resident adult and subadult squirrels in the analyses. We included 78 seasonal (summer and fall) home ranges of 35 red squirrels (15 male, 20 female) and 41 seasonal home ranges of 26 Abert’s squirrels (17 male, 9 female) in our analyses. Depending on locations of the occupied middens and where Abert’s squirrels were captured, selected home ranges may encounter either roads or random lines or both.

For each species, we used generalized linear mixed modeling (GLMM) with a logit link function and binomial error distribution to compare the probability of total or core home range that included two types of linear features (roads and random lines) with ‘include’ as a binary response variable (included in the home range = 1, did not include in the home range = 0, Fig 1b and 1c). We then compared the odds of including roads in the home ranges with the odds of including random lines. We used linear mixed effect models (LMM) to compare distance from the center of total or core home ranges to the linear features. For both GLMM and LMM, We included types of linear features (roads or random lines), sex, and season (summer or fall) as fixed effects, and individual squirrels as a random effect. If squirrels avoided roads, number of home ranges that included roads will be lower than those that included random lines, and distance from the center of home ranges to roads should be greater than distance to random lines.

### Effects of roads and traffic on movements

#### Traffic monitoring

We used bi-directional traffic counters (TRAFx Vehicle Counter Model G3, TRAFx Research Ltd, Canmore, Alberta, Canada) to record number of vehicles. Traffic counters were placed at roadsides for each road and set to a slow rate, 3 s delay, and 014 threshold of sensitivity with time stamp mode that records time when vehicles were detected. Traffic data were summarized into hourly and daily total traffic volume for each road. Mean hourly traffic volume was 5.33 vehicles (SE 0.18) on the high traffic road (*n* = 429), 2.37 vehicles (SE 0.17) on the medium traffic roads (*n* = 230), and 0.54 vehicles (SE 0.22) on the low traffic road (*n* = 266).

#### Animal movement paths

To collect movements of squirrels near roads, we selected red squirrel occupied middens <100 m from roads and Abert’s squirrels that were captured <200 m from roads. We located each squirrel 2 to 5 times per hour from 8:00 to 18:00 by biangulation or homing. We minimized disturbances by maintaining a distance of >5 m between trackers and animals. When squirrels moved, we did not follow squirrels immediately but waited for 5 min to start tracking. When squirrels were determined to be near on the basis of strength of radio signal, we moved slowly and carefully to prevent encountering squirrels accidentally. Locations were removed if squirrels appeared to be disturbed by surveyors, but this situation rarely occurred. We collected 639 hours of movement data (2685 locations, 218 individual daily movement paths) from 22 red squirrels (10 male, 12 female) and 382 hours of movement data (1753 locations, 91 individual daily movement paths) from 15 Abert’s squirrels (6 females, 9 males). The proportion of homing locations was 75% for red squirrels and was 80% for Abert’s squirrels. We used ArcMap 9.3 to plot squirrel locations, calculate distance from roads, and record the number of road crossings per hour. We used Hawth’s Tools [61] to convert locations into individual daily movement paths and calculated movement parameters for each step, including step length (m)-the distance between the current point and the next location, net displacement (m)-the distance between the first location in the path and the current location [61], duration (min)-time between the current point and the next location, speed (m/min) and tortuosity (step length/net displacement)-a unitless numerical description of the sinuosity (i.e. departure from linearity).

#### Simulation of movement paths

**Fig 2:**
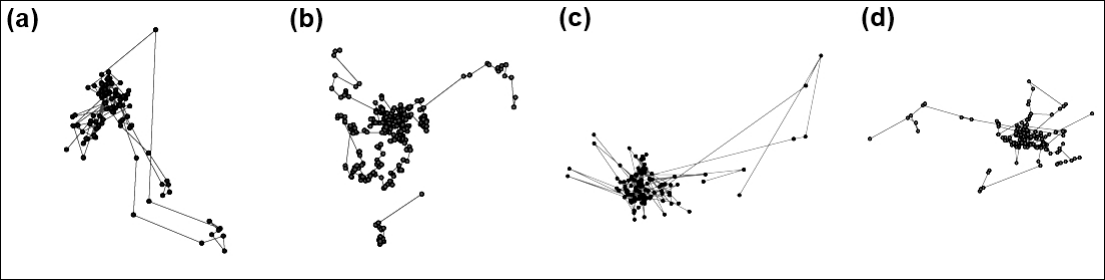
Examples of an observed daily movement path and a simulated correlated random walk of Abert’s squirrels (*Sciurus aberti*) and Mt. Graham red squirrels (*Tamiasciurus hudsonicus grahamensis*) on Mt.Graham, Arizona, USA. (a) Abert’s squirrels-observed, (b) red squirrels-observed, (c) Abert’s squirrels-simulated, (d) red squirrels-simulated.

To assess whether squirrels crossed roads less than expected by chance, we compared the rate of road crossing on the basis of observed movement paths with simulated individual-based correlated random walk (CRW [62]) models (Fig 2). For each observed daily movement path, we used GME to construct a simulated movement path with the same starting location and number of steps as the observed path. The simulated path was created by making independent random draws from the distribution of step length and turn angle based on the empirical distributions. The distribution of observed turn angle and step length was likely affected by roads and traffic. To minimize influence of roads and traffic, for each simulated CRW, we built the distribution of turn angle and step length based on observed locations that were not near roads (distance to roads was >25% quartile of the observed distribution of distances from squirrel locations to roads).

#### Data analysis

We summarized squirrel movement paths into hourly records (total step length, mean tortuosity, mean speed, mean distance from roads, number of road crossing) and calculated the mean for individual squirrels. We used *t*-tests to compare distance moved per hour, distance from animal locations to roads, rate of road crossing, speed, and path tortuosity between red squirrels and Abert’s squirrels. Although number of locations per hour varied, median time between consecutive locations was 12 min (SE 0.4; 25% quartile: 10 min; 75% quartile: 18 min). As a result of the narrow distribution of times, and the slow travelling speed of squirrels, we feel confident that the comparisons are appropriate.

We used a paired *t*-test to determine whether the mean observed hourly rate of road crossing by squirrels differ from rate of road crossing of simulated random walks. To assess whether animals moved differently when crossing roads, we selected steps before, during, and after road crossings and used an analysis of variance (ANOVA) to compare speed among steps. We used ANOVA to compare hourly rate of road crossing per squirrel among low to high traffic roads. To investigate whether squirrels crossed roads during periods of low traffic volume, we used one-sample *t* tests to compare hourly traffic volume when squirrels crossed roads to the mean hourly traffic of roads. To investigate effects of roads and traffic on squirrel movements, we selected records with total step length >0 m, and used LMM to determine influences of hourly traffic volume on tortuosity, speed, and distance from squirrel locations to roads. We also used LMM to evaluate effects of distance to roads on path tortuosity and speed. We included individual squirrels as a random effect in all models. For red squirrels, we also included distance from occupied middens to roads in models as a fixed effect. To meet assumptions of normality, we applied a natural log (ln) transformation to tortuosity and speed, and a square root transformation to distance to roads. We ran LMM with the ‘lme4’ (Linear mixed-effects models using Eigen and S4 [63]) package in R (version 3.1.0 -"Spring Dance", R Development Core Team 2014).

## Results

### Effects of roads on animal space use

**Fig 3:**
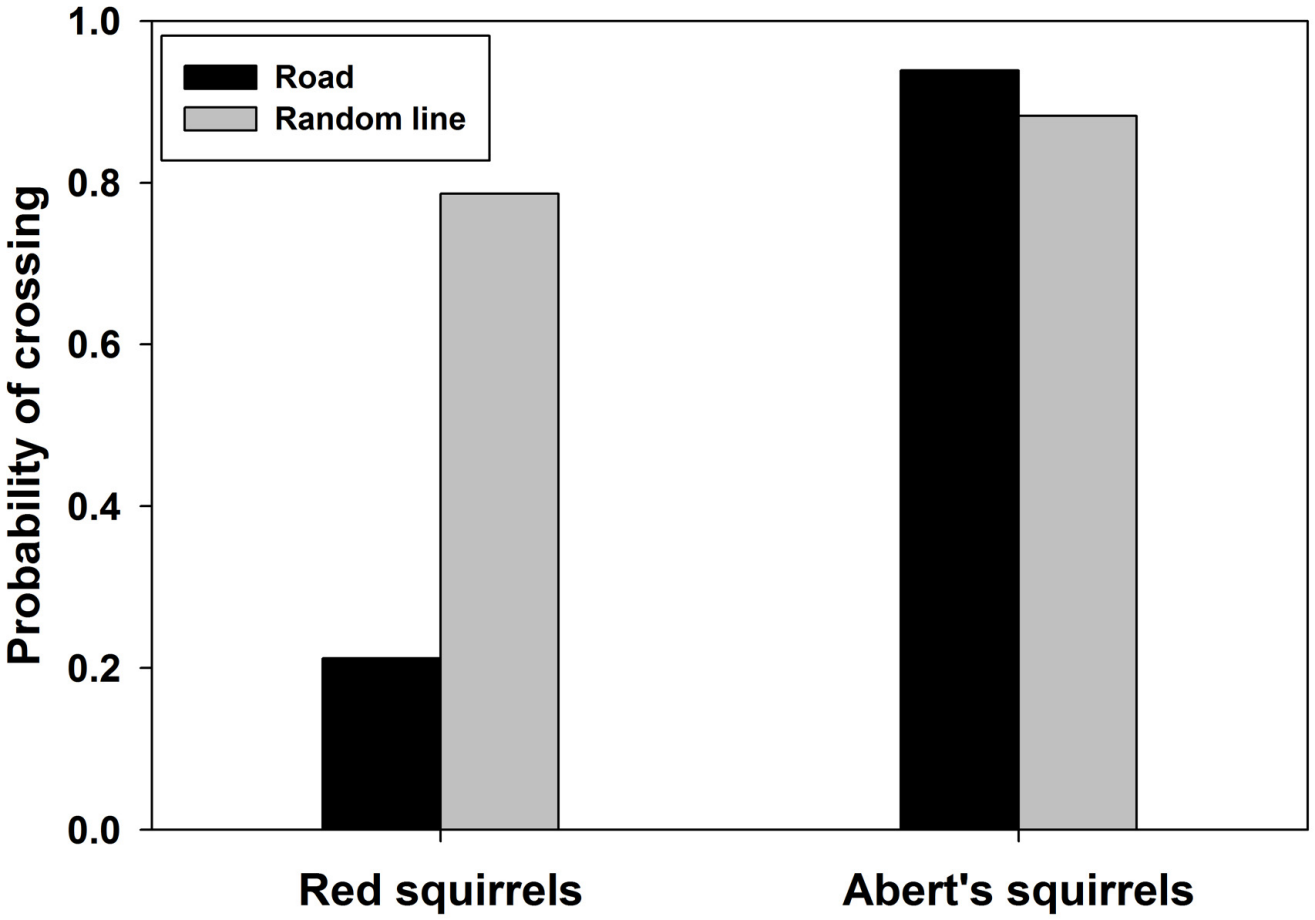
Probability of 95% fixed kernel home ranges of Mt. Graham red squirrel (*Tamiasciurus hudsonicus grahamensis*) and Abert’s squirrels (*Sciurus aberti*) that include roads and random lines in forests on Mt. Graham, Arizona, USA.

Red squirrels avoided roads, as odds of including random lines in home ranges were 13.7 times of odds of includingroads (Table 1, Fig 3) and distance from the center of total home ranges to roads (71.2 m [SE 5.0]) was greater than distance to random lines (51.5 m [SE 5.0], *t*_96_ = 3.07, *p* = 0.002). In contrast, Abert’s squirrels tended to approach roads, as odds of including roads were 2.0 times of odds of including random lines (Table 1, Fig 3), and distance from the center of total home ranges to roads (89.0 m [SE 10.95]) was similar to distance to random lines (116.2 m [SE 10.96], *t*_81_ = -1.09, *p* = 0.28). Red squirrels avoided areas adjacent to roads, as indicated by lower odds of including roads in their core home ranges compared to random lines (Table 1), although distance from the center of core home ranges to roads (60.9 m [SE 4.7]) was similar to distance to random lines (52.8 m [SE 4.6], *t*_95_ = 1.32, *p* = 0.18). On the other hand, Abert’s squirrels did not avoid roadside areas, since odds of including roads in their core home ranges were similar to including random lines (Table 1), as well as distance from the center of core home ranges to roads (94.6 m [SE 11.1]) and to random lines (123.4 m [SE11.12], *t*_980_ = -1.06, *p* = 0.29).

**Table 1:** Estimated coefficients of generalized linear mixed models for probability of 95% and 50% fixed kernel home ranges of Mt. Graham red squirrels (*Tamiasciurus hudsonicus grahamensis*) and Abert’s squirrels (*Sciurus aberti*) including roads and random lines in forests, 2008–2012, Mt. Graham, Arizona, USA.

	95% Kernel			50% Kernel		
Variables	Estimate	SE	*P*	Estimate	SE	*P*
**Red squirrels**						
**Roads (random lines as reference)**	-1.31	0.73	0.02	-26.07	7.22	<0.001
**Season: Summer (fall as reference)**	1.86	0.68	0.27	15.65	4.78	0.001
**Sex (Male)**	-0.57	0.87	0.38	-0.86	3.70	0.82
**Abert’s squirrels**						
**Roads (random lines as reference)**	2.73	1.19	0.06	-0.40	0.47	0.37
**Season: Summer (fall as reference)**	-1.11	1.00	0.006	-0.30	0.50	0.55
**Sex (Male)**	0.87	0.99	0.51	0.41	0.48	0.40

### Effects of roads and traffic on movements

Mean distance traveled per hour was not different between red squirrels (104.27 m [SE 10.58]) and Abert’s squirrels (92.18 m [SE 12.51], *t*_34_ = -0.74, *p* = 0.47), nor was mean distance to roads (red squirrels: 82.08 m [SE 9.87]; Abert’s squirrels: 92.7 m [SE 11.7], *t*_34_ = 0.69, *p* = 0.49). Red squirrels moved at similar speed to Abert’s squirrels (*t*_34_ = -0.79, *p* = 0.44). Mean speed of red squirrels and Abert’s squirrels was 2.3 m/min (SE 0.4) and 1.8 m/min (SE 0.5) respectively. Mean tortuosity of movement paths was 1.6 (SE 0.3) for red squirrels, which tend to be lower than tortuosity of movement paths of Abert’s squirrels (2.3 [SE 0.3], *t*_33_ = 1.85, *p* = 0.07).

**Fig 4:**
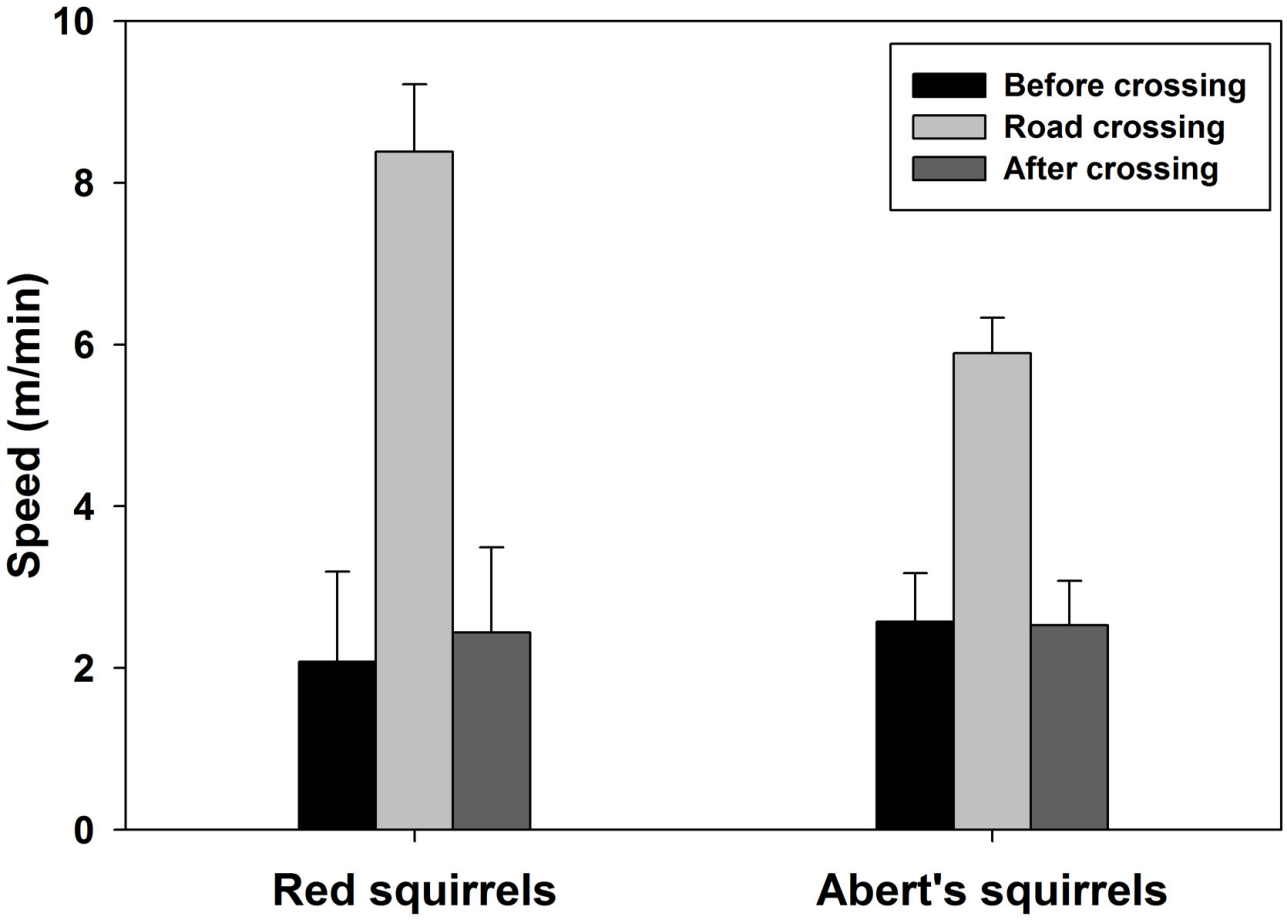
Mean speed before, during, and after road crossings by Mt. Graham red squirrel (*Tamiasciurus hudsonicus grahamensis*) and Abert’s squirrels (*Sciurus aberti*) on Mt. Graham, Arizona, USA.

Eleven of 22 red squirrels (5 male, 6 female, 50%) and 11 of 15 Abert’s squirrels (6 male, 5 female, 73.33%) crossed roads at least once. Mean rates of road crossing by Abert’s squirrels (0.15 crossings/h [SE 0.04]) did not differ from rates of road crossing by red squirrels (0.09 crossings/h [SE 0.04], *t*_34_ = 1.07, *p* = 0.29). Red squirrels and Abert’s squirrels crossed roads less often than that of the random walk models (red squirrels: *t*_21_ = -2.05, *p* = 0.05; Abert’s squirrels: *t*_14_ = -2.20, *p* = 0.05). Both red squirrels and Abert’s squirrels increased speed during road crossing compared to before and after road crossings (red squirrels: *F*_2,117_ = 14.45, *p* < 0.001; Abert’s squirrels *F*_2,117_ = 15.54, *p* < 0.001, Fig 4). Red squirrels moved faster than Abert’s squirrels during road crossings (*t*_122_ = -2.15, *p* = 0.03). Mean speed of road crossing by red squirrels and Abert’s squirrels was 8.38 m/min (SE 0.41) and 5.89 m/min (SE 0.44) respectively.

**Fig 5:**
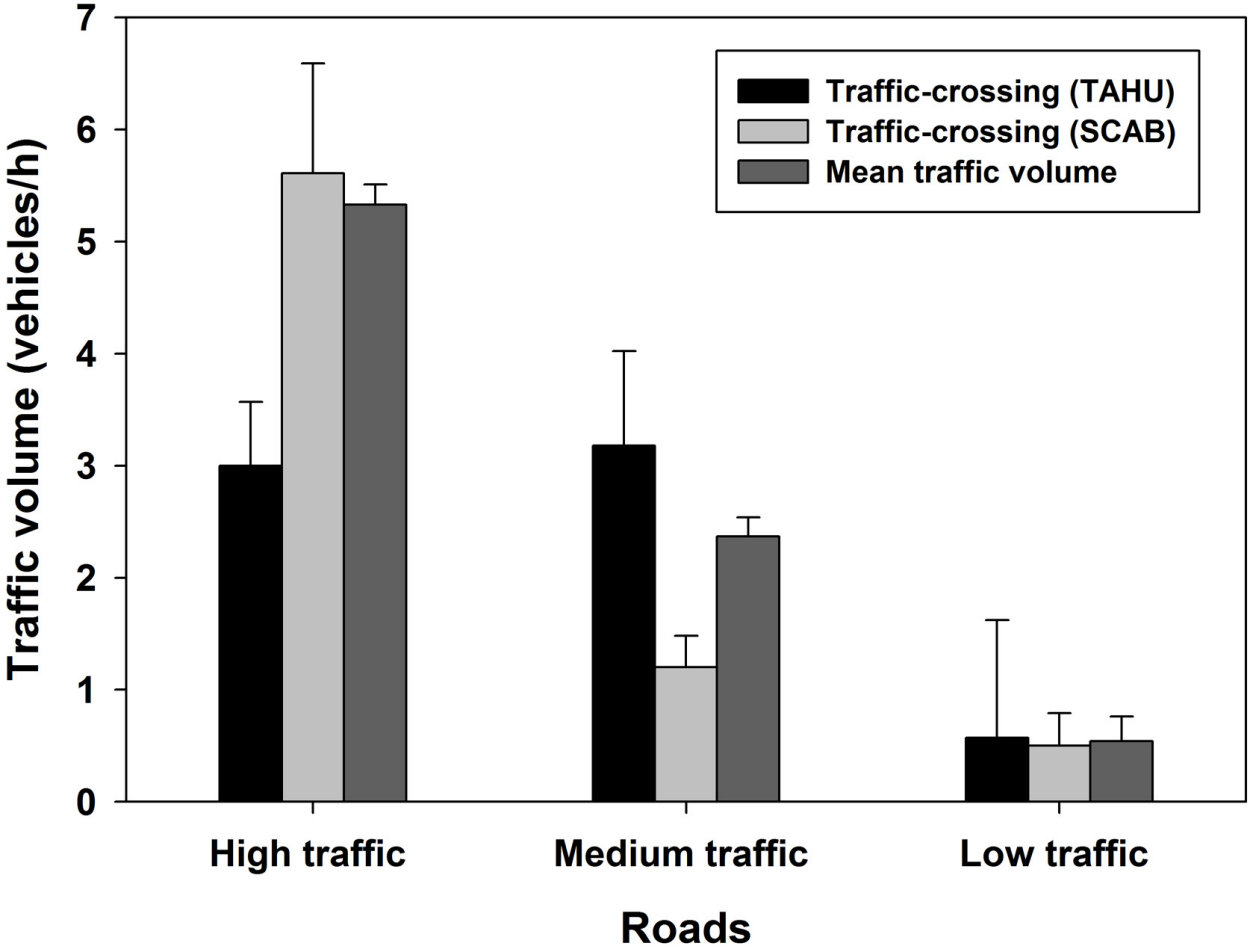
Traffic volume during road crossings by Mt. Graham red squirrel (*Tamiasciurus hudsonicus grahamensis*, MGRS) and Abert’s squirrels (*Sciurus aberti*, SCAB), Mt.Graham, Arizona, USA on Mt. Graham, Arizona, USA.

Hourly rate of road crossings per squirrel was not different among low to high traffic roads for both species (red squirrels: *F*_2, 20_ = 2.29, *p* = 0.13; Abert’s: *F*_2, 22_ = 0.55, *p* = 0.57). Mean traffic volume when red squirrels crossed roads was lower than the mean hourly traffic volume on the high traffic roads (*t*_23_ = -4.59, *p* < 0.001) but was similar on the medium traffic (*t*_10_ = 0.69, *p* = 0.51) and low traffic roads (*t*_5_ = 0.07, *p* = 0.94, Fig 5). Mean traffic volume when Abert’s squirrels crossed roads did not differ from the mean hourly traffic volume on the high traffic (*t*_2_ = 0.29, *p* = 0.77) and low traffic roads (*t*_3_ = -0.13, *p* = 0.90) but was lower on the medium traffic roads (*t*_14_ = -4.19, *p* < 0.001, Fig 5). Red squirrels traveled slower when near roads, and path tortuosity increased as hourly traffic volume increased (Table 2). Speed of Abert’s squirrels was not affected by roads and traffic, but path tortuosity increased as distance to roads increased (Table 3).

**Table 2:** Effects of traffic volume and distance to roads on Mt. Graham red squirrel (*Tamiasciurus hudsonicus grahamensis*) movement, Mt.Graham, Arizona, USA.

Variables	Distance to roads			Speed (m/min)^b^			Path tortuosity^c^		
	Estimate	SE	*P*	Estimate	SE	*P*	Estimate	SE	*P*
**Traffic volume (vehicles/h)**	0.02	0.03	0.41	0.00	0.00	0.65	0.02	0.007	0.06
**Distance to roads (m)**^a^	NA	NA	NA	0.03	0.01	0.008	-0.01	0.01	0.42

^a^Distance to roads was square root transformed

^b^Speed was natural-log transformed

^c^Path tortuosity (net displacement/step length) was natural-log transformed

**Table 3:** Effects of traffic volume and distance to roads on Abert’s squirrels (*Sciurus aberti*) movement, Mt.Graham, Arizona, USA.

Variables	Distance to roads			Speed (m/min)^b^			Path tortuosity^c^		
	Estimate	SE	*P*	Estimate	SE	*P*	Estimate	SE	*P*
**Traffic volume (vehicles/h)**	0.04	0.05	0.37	0.02	0.01	0.07	0.00	0.01	0.63
**Distance to roads (m)**^a^	NA	NA	NA	0.00	0.01	0.89	0.04	0.01	0.003

^a^Distance to roads was square root transformed

^b^Speed was natural-log transformed

^c^Path tortuosity (net displacement/step length) was natural-log transformed

## Discussion

### Forest-dependent species and edge-tolerant species responded to roads differently

Life history traits of species are critical in assessment of effects of linear development on species persistence. Mammals with greater mobility, lower reproductive rates and larger body sizes are more vulnerable to the negative effects of roads and traffic on animal abundance or density [24]. However, road impacts on animal populations also depend on species-specific behavioral responses to roads [8,64]. We demonstrated species with similar body size (< 1 kg) that differ in habitat preference and foraging strategy responded to roads quite differently. Such behavioral and ecological variation is important when considering effects of roads on wildlife. Our results indicate that roads restricted movements and space use of a native forest-dependent species while creating habitat used by an exotic edge-tolerant species. Road edges differ from natural edges or edges produced by clearcuts in their linear configuration, length, and spatial extensive effects driven by associated anthropogenic disturbance [5,14,65]. Consequently, forest fragmentation and edges introduced by roads are widely distributed, tend to exist for long periods of time and are exacerbated by frequent disturbance [65–67]. Species with similar habitat preference or foraging strategy tend to respond to human disturbance in similar fashion [68,69]. Species that avoid open ground, such as the cotton rat (*Sigmodon hispidus*), prairie vole (*Microtus ochrogaster*), San Diego pocket mouse (*Chaetodipus fallax*) and most forest-dependent insectivorous birds avoid crossing narrow dirt roads [68,70–72]. Alternatively, species that prefer open areas such as edge-tolerant forest bats (e.g. *Barbastella barbastellus*), Dulzura kangaroo rat (*Dipodomys simulans*), and yellow-necked mouse (*Apodemus flavicollis*) are more likely to cross roads and use roads as movement routes [72–74].

### Effects of roads on animal space use

Roads and traffic can serve as barriers that impede animal movements, decrease accessibility of resources such as food, shelter or mates, lead to reduction in reproductive success and gene flow, and ultimately threaten population persistence [7,75,76]. Barrier effects of roads have been documented in a diversity of terrestrial fauna, including insects, reptiles, amphibians, birds and mammals [68,77–80]. Avoidance of roads by red squirrels was suggested previously through live trapping along culverts, as red squirrels are scarce at culverts despite being the most abundant species in the adjacent forest [81]. Animals may avoid roads because of vehicles, traffic disturbance, environmental changes at roadside areas, and gap of understory or canopy cover created by roads [9,64,68,82]. Forest specialists often avoid entering gaps with low canopy or understory cover, and hence are especially vulnerable to habitat fragmentation and barrier effects of roads [10,30]. Contrarily, roads enhance dispersal of species that exist mainly in open areas [83]. Cane toads (*Bufo marinus*) move faster along roads than in more densely vegetated areas and actively favor roads as movement corridors [15]. Because Abert’s squirrels are relatively edge-tolerant compare to red squirrels, likely because of their larger body size, terrestrial locomotion, and preference of nest sites with less closed canopy [33,35,52], it is not surprising that Abert’s squirrels did not avoid roads. Although Abert’s squirrels were more likely to include roads in their home ranges compared to random lines in forests, the rate of road crossing was lower than null models of correlated random walks. This suggests that edge-tolerant species may select roadside areas over forest interior, but movements may still be impeded by roads and traffic. For example, eastern chipmunks (*Tamias striatus*) are common near roads but rarely cross roads spontaneously [84]. Despite a preference for areas with moderate road density because of association of roads and deciduous forest [27], moose (*Alces alces*) avoid individual roads [85].

Animal populations and distribution are influenced by environmental changes in forest structure, microclimate, and forest dynamics near road edges, including lower forest density, increased solar radiation, wind velocity and light availability, extreme temperature, and predominately edge-adapted plant species [17,67]. We demonstrated red squirrels selected forest interior over roadside areas and Abert’s squirrels showed no preference between these two. Red squirrels select middens with higher basal area and higher canopy cover [34,40], likely due to higher protection from avian predators and greater food production [29,86]. Promotion of open forests is used to increase abundance of Abert’s squirrels [87], suggesting that Abert’s squirrels may be more capable to use the open areas introduced by roads. Roads lead to greatly reduced habitat availability of species that avoid roads [14]. For instance, grizzly bears (*Ursus arctos*) use habitat within 100 m of roads less than expected [88]. Conversely, black vultures (*Coragyps atratus*) and turkey vultures (*Cathartes aura*) prefer areas with greater road density [89]. Because of differential responses of red squirrels and Abert’s squirrels to roads, the distribution and population of these two species is likely to be affected by roads.

### Effects of traffic and roads on animal movements

Effects of traffic intensity on animal movements are difficult to disentangle from the influence of road characteristics, because of temporal variation in traffic volume and positive correlation with road width [31,32]. We addressed the issue by examining effects of concurrent traffic volume on animal movements directly instead of comparison between high and low traffic roads. We demonstrated that squirrels crossed medium and high traffic roads during low traffic periods, that resulted in similar rates of road crossing among roads with different traffic volumes. Moreover, we revealed that roads and instantaneous traffic intensity influence animal movement patterns and provided hints of how animals perceive traffic disturbance. Red squirrels and northern flying squirrels (*Glaucomys sabrinus*) pause more often and reduce moving speed across clearcuts, compared to traveling less direct routes through forests after translocation [90,91]. However, we found both Mt. Graham red squirrels and Abert’s squirrels increased speed when crossing roads spontaneously in familiar environment. Red squirrels move at relatively slow speeds with frequent stops after transfer away from home to unfamiliar ground [92]. Therefore, we hypothesize that reduced speed during gap crossing by squirrels in previous studies (i.e. [90,91]) may be a response to unfamiliar environment rather than to clearcuts. Changes in movement patterns such as speed and posture during road crossing are reported in other species. Moose increase speed before, during, and after highway crossings [85]. Grizzly bears move faster near roads [93]. Hedgehogs (*Erinaceus europaeus*) run with legs extended to raise the belly above the ground when crossing roads, which is different from their posture during foraging [94]. Tree squirrels rely on canopy cover to provide shelter and use arboreal escape routes when encountering aerial or ground predators [95]. Red squirrels are more likely to run from tree to tree when tree density is low [92]. We observed that Mt. Graham red squirrels tend to travel in trees before descending to the ground for road crossing, and climb trees immediately after crossing. On Mt. Graham, the major source of mortality in red squirrels is avian predation [56], and mortality is higher in relatively open forests [41]. As a result, increased speed by squirrels during road crossings is likely to reduce risk of predation.

Animals often perceive anthropogenic disturbance as a threat, with corresponding decreases in foraging time and increased stress response [96], but how human disturbance affects animal movements varies by species. Slow speed and increased path tortuosity may indicate high quality habitat. For example, cougars (*Puma concolor*) travel slowest through riparian areas with vegetation types that are highly preferred and fastest through human-dominated areas that are low quality habitats [97]. Alternatively, animals may also travel slower with increased path tortuosity under risk of predation and human disturbance. Venomous snakes cross roads more slowly and cease movement in response to passing vehicles [98]. Path tortuosity of gray wolves increases at areas of high-trail and road density [99]. The risk of depredation is higher near linear infrastructure [100]. We suggest red squirrels may perceive traffic disturbance as a threat similar to predators and are exposed to higher predation risk near roads. The behavioral responses of reduced speed near roads and increased tortuosity of movement paths with increased traffic volume resemble responses to greater predation risk [90,96,99]. Conversely, Abert’s squirrels appeared little affected by roads and traffic with tortuosity of movement paths reduced as distance to roads decreased.

### Roads may affect interactions between native and exotic species

Coexistence of potentially competing species is possible by niche differentiation [101–103]. Nonetheless, on Mt. Graham, habitat use and dietary overlap is high between red squirrels and Abert’s squirrels [48,49]. Moreover, Mt. Graham red squirrels had not recently experienced competition with other tree squirrels because of >10,000 years of isolation and may become more vulnerable to competitive exclusion from Abert’s squirrels [51,104]. Abert’s squirrels consume as much as 74% of annual cone crops in their native ranges [50]. On Mt. Graham, Abert’s squirrels remove cones faster than red squirrels and reduce cone availability for red squirrels [105]. Even though Abert’s squirrels may have higher risk of mortality due to wildlife-vehicle collisions because of little road avoidance, negative effects of exotic Abert’s squirrels on native red squirrels via resource exploitation competition may be exacerbated by roads and traffic disturbance. Because mean home range size of Abert’s squirrels was three times larger than that of red squirrels, one Abert’s squirrel can cross roads and affect several red squirrels whereas red squirrels’ ability to collect food or chase intruders are constrained by roads and traffic. Throughout the British Isles and northern Italy, exotic eastern gray squirrels (*S*. *carolinensis*) cause reduced energy intake for native Eurasian red squirrels (*S*. *vulgaris*) by competition for food that result in reduced recruitment and population size [13,106]. Part of the success of alien eastern gray squirrels may be the ability to persist in a heavily fragmented landscape [30]. Whereas forest-dependent species usually show negative response to roads [68], edge-tolerant species that are less sensitive to habitat fragmentation may benefit from roads and displace forest interior species [36]. Proportion of edge species increases substantially within 100 m of roads and differs from adjacent communities [83]. Research that investigates how interspecific differences in behavioral responses to roads may influence species interactions and population persistence is warranted.

### Conservation Implications

Abert’s squirrels have been removed by recreational hunting on Mt. Graham; however, the success of removal may be limited [107]. To improve road permeability for endangered native red squirrels while minimizing risk of mortality due to wildlife-vehicle collisions, we suggest canopy closure be maintained at roadside areas and perhaps install canopy bridges with cover [108] and small entrances that cannot be entered by exotic Abert’s squirrels. The global human population has exceeded 7 billion in 2012 [109] and is projected to grow to 9.6 billion by 2050 [2]. Anthropogenic development undoubtedly poses the greatest challenge to reconcile the pursuit of economic growth with the protection of ecological integrity of wildlife habitats [67,110]. Road networks and traffic intensity are continuing to increase worldwide, especially in the Asia Pacific region [110,111]. Roads in India (4.6 million km [6]) increased 78% in 40 years and 20,000 km of new and upgraded roads are expected to be completed in the next few years [110]. The ecological and genetic consequences of inhibition of movements and population isolation can be serious, particularly for populations of species limited by habitat availability or at the edge of their distribution range [112,113]. Species are rarely endangered by a single cause and 17% of the species endangered by roads are simultaneously impacted by non-native species in the United States [3]. Understanding the effects of human disturbance on endangered wildlife populations is critical to conservation. Documenting the response of red squirrels and Abert’s squirrels to roads and traffic has important conservation implications as both species can serve as models for other forest species with similar habitat preference in forest density and foraging strategy. Forest ecosystems worldwide have been fragmented excessively through human activities, and the degree of fragmentation is exacerbated by continually increasing demand for outdoor recreational activities and development [114,115]. Our study provides insight to animal responses to anthropogenic development and disturbance, and helps to anticipate how the expanding transportation network may impact animal abundance, population density, and persistence.

## Supporting Information

S1 Dataset Data used in analyses. (ZIP)
